# Prevalence and impact of migraine among university students in Bangladesh: findings from a cross-sectional survey

**DOI:** 10.1186/s12883-022-02594-5

**Published:** 2022-02-26

**Authors:** Abdur Rafi, Saiful Islam, M. Tasdik Hasan, Golam Hossain

**Affiliations:** 1 Rajshahi Medical College, Rajshahi, 6000 Bangladesh; 2 Department of Public Health and Informatics, Jahangirnagar University, Savar, Dhaka Bangladesh; 3 Youth Research Association, Savar, Dhaka Bangladesh; 4 Department of Primary Care & Mental Health, University of Liverpool, Liverpool, UK; 5 Health Research Group, Department of Statistics, University of Rajshahi, Rajshahi, 6205 Bangladesh

**Keywords:** Migraine, ID Migraine™, HIT-6, Uiversity students, Bangladesh

## Abstract

**Background:**

Migraine is one of the main causes of long-term morbidity, and it is one of the major contributors of all types of headaches in worldwide. Despite its disruptive effect, it is frequently underdiagnosed and undertreated in Bangladesh. The aim of the present study was to determine the prevalence of migraines and its impact on daily life of university students in Bangladesh.

**Methods:**

This cross-sectional study was conducted among 2,352 students of Rajshahi University and Jahangirnagar University during March 2020 through a self-administered online survey. ID Migraine™ scale and HIT-6 scale were used to screen migraine and its impact respectively. Frequency distribution, Chi-square test and t-test along with multiple logistic regressions model were used to determine the prevalence and associated factors of migraine respectively.

**Results:**

The overall prevalence of migraine among the participants was 21.4%. The prevalence was higher among females (29%) than males (12%). A multivariable logistic regression model provided the following eight risk factors of migraine: (i) gender (*p* < 0.01), (ii) family income (*p* < 0.01), (iii) marital status (*p* < 0.01), (iv) infrequent exercise (*p* < 0.01), (v) family history of headache (*p* < 0.01), (vi) high screen time (*p* < 0.05), (vii) depressive symptoms (*p* < 0.05) and (viii) anxiety disorder (*p* < 0.01). More than two-thirds of the people with migraines reported more than five attacks during the past month with moderate to severe intense headache. Stress was the most reported trigger of migraine among university students (71%) followed by irregular sleep (47%), academic pressure (33%), and external noise (28%). Almost 37% of the participants who had migraines reported that headache caused severe impact in their day to day life.

**Conclusions:**

The prevalence of migraine among university students of Bangladesh is alarmingly high. Frequent migraine attacks and severe intensity of headache cause a substantial level of impact among the sufferers. Cautious avoidance of the triggering factors through appropriate interventions and prophylactic medication can mitigate the negative impact of migraine as well as improve the quality of life.

## Background

Headache disorder is one of the main causes of long-term morbidity worldwide. It is listed as the second leading cause of years lived with disability for the last three decades according to the Global Burden of Disease Study [1]. Migraine is one of the major contributors of all types of headaches with a lifetime prevalence of 14% to 16% around the world [2]. It is often associated with impaired social and professional life and reduced productivity, making it responsible for roughly 3% of disability making it the eighth most burdensome disease [2, 3].

A wide range of factors including stress, noise and sound, fatigue, fasting, sleep disorder, alcohol drinking, etc. are associated with the precipitation of migraine [4]. These triggering factors notably educational stress and irregular sleep are more prevalent among young adults compared to other age groups, especially university students, which have made them vulnerable to migraines. It is reported that almost 10–18% of university students suffer from migraine worldwide [5]. This high prevalence of migraine impairs the academic performance of the students and also decrease the quality of life of the sufferers [6, 7].

Regardless of its disruptive effect, migraine is frequently underdiagnosed and undertreated in Bangladesh. There is scarcity of evidence reporting the prevalence and effect of migraine among university students of Bangladesh. A study including a small sample reported that the prevalence of migraines as 17.4% among medical students suffering from irritable bowel syndrome [8]. However, the study did not evaluate the risk factors and the effect of migraine on their life events. University students are asset of a particular country, special attention is paid to university students considering their potential contribution to the nation. Due to their unique role in the country, it is important to understand the prevalence and identify the risk factors of migraine as well as its effect on their daily life for taking further preventive measures.

Therefore, the present study aims to determine the prevalence and risk factors of migraines among the university students of Bangladesh as well as the effect of this disease on their daily life.

## Methods

### Study design and setting

This was a cross-sectional study conducted among the two selected universities students (i.e., University of Rajshahi and Jahangirnagar University) during March 2020 through an online survey. The University of Rajshahi and Jahangirnagar University is the second and fourth largest universities in Bangladesh respectively, with students coming from the different parts of the country. A total number of 55,276 students are studying in these two universities (38,495 in University of Rajshahi, and 16,781 in Jahangirnagar University).

### Sample size calculation and sampling method

The sample size was calculated from the prevalence estimate using the formula: $\mathrm{n}=\frac{{\mathrm{z}}^{2}\mathrm{pq}}{{\mathrm{d}}^{2}}$, where, n = number of the samples; z = 1.96 for 95% confidence interval (CI), *p* = “best guess” for prevalence and d = precision of the prevalence estimate. We did not find any existing data on the prevalence of migraine among the university students of Bangladesh. However, a previous study from the neighboring country India reported the prevalence as 14.12% [9], which was considered as p (best guess) value (*p* = 0.1412) for calculating sample size for the present study, and the formula provided that 2337 sample would be the required size. Assuming a 10% non-response rate, a total of 2600 university students were approached. The convenient sampling method was used to include the participants in this study as those who had the social media id and personal relation with the recruited volunteers had the chances of enrollment in the study.

### Data collection procedure

A self-administered online survey form created in Google forms were used to collect data from the participants. The survey link was posted in a regular interval of one week in the internal social media groups of the university students and an open request was placed by the team of investigators to fill-up the form. Also, 20 volunteers from different departments of these universities were employed to circulate the survey link among their student networks, in addition to regular posting in the above-mentioned social media groups. They were instructed to be inclusive, open, and circulate it periodically for maximum reach. Login with email and providing student ID number was mandatory for limiting single response. Email addresses of the participants were collected upon proper clarification and informed consent for the reliability of the data. The study was conducted following the Checklist for Reporting Results of Internet ESurveys (CHERRIES) guidelines [10].

### Data collection instruments

A self-developed questionnaire was used to collect information from patients. The survey questionnaire comprised of four parts: (i) socio-demographics, lifestyle and behavioral factors related data, (ii) headache-related data, (iii) impact of headache, if migraine was present using the Headache Impact Test (HIT-6), and (iv) presence of anxiety and depressive symptoms using two psychometric scales (the GAD-7 for assessing anxiety disorder and the PHQ-9 for assessing depressive symptoms). As the ID Migraine™ and HIT-6 scales were not used before among Bangladeshi population, these were not validated in Bangla. We have used back translation method for translating the tools after proper consent from the developers under supervision of a team of three consultant neurologists of the Department of Neuromedicine of RMCH (Rajshahi Medical College & Hospital). A pretest of the questionnaire was done in the Department of Neuromedicine, RMCH among 30 diagnosed migraine patients by a consultant neurologist.

### Part 1: Socio-demographics, lifestyle and behavioral factors

Socio-demographic information was collected during the survey by asking questions concerning age, gender, study year, monthly family income, marital status, height, and weight. Lifestyle and behavioral factors included fast food intake (frequency per week), amount of physical exercising (days per week for at least 30 min a day), smoking habits (yes/no), alcohol intake (yes/no), substance abuse (yes/no), and sleep quality measured by the Pittsburgh sleep quality index (PSQI) which is an appropriate screening tool for measuring sleep dysfunction in both clinical and non-clinical samples. The PSQI score was categorized as poor (PSQI score > 5) and good (PSQI score ≤ 5) [11].

### Part 2: Headache related data

Participants were initially evaluated by the question “*Did you have two or more headaches in the last 3 months?*” Those who responded ‘yes” were considered as the subjects with potentially troublesome headaches and further screened using the ID Migraine™ test. The screening question was adopted from previous evidence [6, 12]. The ID-Migraine™ test is a three-item self-administered screening tool, developed by Lipton et al. (2003) [13]. It consists of three questions regarding problems related to migraines over the last three months: 1. *Did you feel nauseated or sick in your stomach with your headaches?* 2. *Did light bother you when you had a headache (a lot more than when you do not have headaches)?* and 3. *Did your headache limit your ability to work, study or do what you needed to do for at least 1 day?* A test-diagnosis of migraine headache is made by at least two positive responses. ID Migraine™ has been validated using the International Classification of Headache Disorders (ICHD) criteria in different studies with a pooled sensitivity of 0.84 and a specificity of 0.76 [14].

Headache related data were collected from the participants who were screened as positive for migraine. These included intensity of headache (measured on a four-point scale where 0 = no headache; 1 = mild headache; 2 = moderate headache; 3 = severe headache recommended for use in migraine research by the International Headache Society) [15], frequency of headache during the past month, associated symptoms of headache, characteristic of headache (unilateral, bilateral, pulsating, and throbbing), frequency of analgesic use during the past month, frequency of healthcare facility visit during past 12 months, migraine triggers, and family history of migraine.

### Part 3: Impact of headache

Headache Impact Test (HIT-6) was used to measure the impact of migraine headaches among the ID Migraine™ positive participants. The HIT-6 is a brief and easy to use instrument, developed by Kosinski et al. (2003) [16] to measure the adverse headache impact and to use in screening and monitoring patients with headaches in both clinical practice and clinical research. It consists of six items regarding problems related to headache (i.e., When you have headaches, how often is the pain severe?) with a five-point Likert scale ranging from 6 (Never) to 13 (Always). The total score was obtained by the summating raw score from each contract ranging from 36 to 78, with greater scores indicating a severe impact. In present study, the headache impact severity was categorized into four classes based on total sores of HIT-6: little or no disability (≤ 49), mild disability (50–55), moderate disability (56–59), and disability (≥ 60). This scale is suggested as a reliable and valid tool for measuring headache impact in migraine [17].

### Part 4: Anxiety and depressive symptoms

*Patient Health Questionnaire (PHQ-9*: The PHQ-9 is one of the most psychometrically sound and robust screening tools, developed by Spitzer et al. (1999) [18] which is one the most widely used instruments for assessing depressive disorder globally including Bangladesh [19, 20]. This scale consists of nine items regarding problems related to depression symptomatology over the past two weeks (e.g., “*Thoughts that you would be better off dead, or of hurting yourself in some way?*”) with a four-point Likert scale ranging from 0 (*Not at all*) to 3 (*Nearly every day*). The total score was obtained by the summating raw score from each contract ranging from 0 to 27. In the present study, those scoring moderate to severe (≥ 10) were classed as having depressive symptoms [21].

*Generalized Anxiety Disorder (GAD-7)*: The GAD-7 is one of the most psychometrically sound and robust screening tools, developed by Spitzer et al. (2006) [22] and used in different countries including Bangladesh for assessing anxiety disorder [23, 24]. The scale comprises seven items regarding problems related to anxiety symptomatology over the past two weeks (e.g., “*Feeling afraid as if something awful might happen?*”) with a four-point Likert scale ranging from 0 (*Not at all*) to 3 (*Nearly every day*). The total score was obtained by the summating raw score from each contract ranging from 0 to 21. In the present study, those scoring moderate to extremely severe (≥ 10) were classified as having anxiety disorder positive [22].

### Statistical analysis

Statistical analysis was conducted using SPSS version 24.0. Descriptive statistics was performed for categorical variables (i.e., frequency and percentage), and for continuous variables (i.e., mean and standard deviation). Chi-square test and t-test were used in case of categorical variables and continuous variables, respectively to investigate the relationship between dependent and independent variables. Binary multiple logistic regressions were performed with a 95% confidence interval to determine the significant associations between categorical dependent and independent variables. Multicollinearity problems among independent variables in multiple logistic model was checked by standard error (SE) suggested by Chan [25]. The association of variables was considered statistically significant if the two-sided *p*-value was less than 0.05.

## Results

### Characteristics of the participants

A total of 2,352 university students participated in the study (response rate 90.5%). Their mean age was 21.9 (SD = 2.3) years. More than half of them (56%) were female. Almost 47% of the participants were from middle income families. BMI of 63% participants was within a normal range (18.5–24.9 kg/m^2^). Among the participants, 25%, 19% and 11% were smokers, alcoholic and substance abusers, respectively. More than 69% of the participants reported poor sleep quality. The prevalence of anxiety and depressive symptoms were 29% and 42%, respectively (Table 1).

**Table 1:** Socio-demographic and lifestyle related characteristics of the participants (*n* = 2352)

Characteristics	Total N (%)	Migraine N (%)	No migraine N (%)	*p*-value
**Age (Mean, SD)**	21.9 (2.3)	21.7 (2.2)	21.9 (2.3)	0.051
**Sex**				
Female	1313 (55.8)	378 (28.8)	935 (71.2)	< 0.001
Male	1039 (44.2)	125 (12.0)	914 (88.0)	
**Year of study**				
1^st^	621 (26.4)	196 (31.6)	425 (68.4)	< 0.001
2^nd^	534 (22.7)	78 (14.6)	456 (85.4)	
3^rd^	511 (21.7)	90 (17.6)	421 (82.4)	
4^th^	403 (17.1)	77 (19.1)	326 (80.9)	
5^th^ / Masters	283 (12.0)	62 (21.9)	221 (78.1)	
**Family income**				
Low (< BDT 15,000)	458 (19.5)	80 (17.5)	378 (82.5)	< 0.001
Middle (BDT 15,000–30,000)	1098 (46.7)	203 (18.5)	895 (81.5)	
High (BDT > 30,000)	796 (33.8)	220 (27.6)	576 (72.4)	
**Marital status**				
Married	261 (11.1)	83 (31.8)	178 (68.2)	< 0.001
Unmarried	2091 (88.9)	420 (20.1)	1671 (79.9)	
**BMI**				
Overweight (≥ 25 kg/m^2^)	434 (18.5)	85 (19.6)	349 (80.4)	0.560
Normal (18.5–24.9 kg/m^2^)	1481 (62.9)	320 (21.6)	1161 (78.4)	
Underweight (< 18.5 kg/m^2^)	437 (18.6)	98 (22.4)	339 (77.6)	
**Frequency of fast-food intake per week**				
More than 3 times	217 (9.2)	45 (20.7)	172 (79.3)	0.840
1–2 times	778 (33.1)	162 (20.8)	616 (79.2)	
Less than once	1357 (57.7)	296 (21.8)	1061 (78.2)	
**Frequency of exercise per week (at least 30 min daily)**				
More than 3 times	423 (18.0)	83 (19.6)	340 (80.4)	0.014
1–2 times	446 (19.0)	118 (26.5)	328 (73.5)	
Less than once	1483 (63.1)	302 (20.4)	1181 (79.6)	
**Screen time (daily)**				
> 12 h	201 (8.5)	51 (25.4)	150 (74.6)	< 0.001
6–12 h	811 (34.5)	223 (27.5)	588 (72.5)	
2–6 h	1052 (44.7)	176 (16.7)	876 (83.3)	
< 2 h	288 (12.2)	53 (18.4)	235 (81.6)	
**Smoking**				
Yes	591 (25.1)	115 (19.5)	476 (80.5)	0.187
No	1761 (74.9)	388 (22.0)	1373 (78.0)	
**Alcohol intake**				
Yes	441 (18.8)	103 (23.4)	338 (76.6)	0.263
No	1911 (81.3)	400 (20.9)	1511 (79.1)	
**Substance abuse (e.g. cannabis, heroine, marijuana, amphetamines etc.)**				
Yes	262 (11.1)	68 (26.0)	194 (74.0)	0.056
No	2090 (88.9)	435 (20.8)	1655 (79.2)	
**Family history of chronic headache**				
Yes	993 (42.2)	303 (30.5)	690 (69.5)	< 0.001
No	1359 (57.8)	200 (14.7)	1159 (85.3)	
**Sleep quality**				
Poor (PSQI score > 5)	1627 (69.2)	388 (23.8)	1239 (76.2)	< 0.001
Good (PSQI score ≤ 5)	728 (31.0)	115 (15.8)	613 (84.2)	
**Anxiety**				
Yes (GAD-7 score ≥ 10)	686 (29.2)	235 (34.3)	451 (65.7)	< 0.001
No (GAD-7 score < 10)	1666 (70.8)	268 (16.1)	1398 (83.9)	
**Depression**				
Yes (PHQ-9 score ≥ 10)	984 (41.8)	282 (28.7)	702 (71.3)	< 0.001
No (PHQ-9 score < 10)	1368 (58.2)	221 (16.2)	1147 (83.8)	

### Prevalence of migraine

The overall prevalence of migraine among the participants was 21.4%. The prevalence was higher among females vs. males (29% vs. 12%), participants from 1^st^ year vs. 2^nd^ year (32% vs. 15%), married vs. unmarried (32% vs. 20%), and participants from high-income families (27.6% vs. 18.5% in middle and 17.5% in low-income families). Moreover, the prevalence of migraine was higher among the participants who reported higher vs. lower daily screen time (27.5% for > 6 h vs. 16.7% for < 6 h), having family history of headache vs. those who hadn’t (30.5% vs. 14.7%), poor vs. good sleep quality (24% vs. 16%), having with vs. without considerable anxiety (34% vs. 16%) and having with vs. without considerable depressive symptoms (29% vs. 16%) (Table 1).

Only the significant factors provided by Chi-square were used as independent variables in multiple logistic regression models, and the magnitude value of SE of each variable was less than 0.50, no evidence of multicollinearity problems among our selected independent variables and the model demonstrated a good fit, Nagelkerke R^2^ = 0.39. After controlling the effect of other variables, the model demonstrated that predictors of suffering from migraine included female sex (aOR 2.53, 95% CI: 1.97–3.24; *p* < 0.01), high family income (aOR 0.70, 95% CI: 0.51–0.96;*p* < 0.05for low income and aOR 0.74, 95% CI: 0.58–0.95; *p* < 0.05 for middle income), being married (aOR 1.54, 95% CI: 1.12–2.11; *p* < 0.01), infrequent exercise (aOR 1.51, 95% CI: 1.14–1.98; *p* < 0.01), high screen time (aOR 1.47, 95% CI: 1.02–2.12; *p* < 0.05) and family history of headache (aOR 2.29, 95% CI: 1.83–2.85; *p* < 0.01). Moreover, those who reported anxiety had 2.35 times higher risk of suffering from migraine (aOR 2.35, 95% CI: 1.77–3.12; *p* < 0.01), while those who reported depressive symptoms had 1.35 times higher risk (aOR1.35, 95% CI: 1.02–1.79; *p* < 0.05) (Table 2).

**Table 2:** Predictors of migraine among the participants in logistic regression model (*n* = 2352)

Characteristics	cOR (95% CI)	*p*-value	aOR (95% CI)	*p*-value
**Sex**				
Female	2.95 (2.36–3.69)	< 0.001	2.53 (1.97–3.24)	< 0.001
Male	Ref		Ref	
**Year of study**				
1^st^	1.64 (1.18–2.28)	0.003	1.46 (0.99–2.12)	0.051
2^nd^	0.61 (0.42–0.88)	0.009	0.63 (0.42–1.05	0.058
3^rd^	0.76 (0.53–1.09)	0.141	0.84 (0.56–1.25)	0.396
4^th^	0.84 (0.58–1.22)	0.369	0.98 (0.65–1.48)	0.946
5^th^/Masters	Ref		Ref	
**Family income**				
Low (< BDT 15,000)	0.55 (0.41–0.73)	< 0.001	0.70 (0.51–0.96)	0.031
Middle (BDT 15,000–30,000)	0.59 (0.48–0.73)	< 0.001	0.74 (0.58–0.95)	0.018
High (BDT > 30,000)	Ref		Ref	
**Marital status**				
Married	1.85 (1.40–2.46)	< 0.001	1.54 (1.12–2.11)	0.007
Unmarried	Ref		Ref	
**Frequency of exercise per week (at least 30 min daily)**				
More than 3 times	0.95 (0.73–1.25)	0.737	1.19 (0.88–1.61)	0.251
1–2 times	1.41 (1.10–1.79)	0.006	1.51 (1.14–1.98)	0.003
Less than once	Ref		Ref	
**Screen time (daily)**				
> 12 h	1.50 (0.97–2.33)	0.065	1.33 (0.82–2.15)	0.244
6–12 h	1.68 (1.20–2.35)	0.002	1.47 (1.02–2.12)	0.041
2–6 h	0.89 (0.63–1.25)	0.504	0.88 (0.61–1.28)	0.525
< 2 h	Ref		Ref	
**Family history of headache**				
Yes	2.54 (2.08–3.11)	< 0.001	2.29 (1.83–2.85)	< 0.001
No	Ref		Ref	
**Sleep quality**				
Poor (PSQI score > 5)	1.67 (1.32–2.10)	< 0.001	1.24 (0.95–1.62)	0.100
Good (PSQI score < 5)	Ref		Ref	
**Anxiety**				
Yes (GAD-7 score > 10)	2.87 (1.37–3.92)	< 0.001	2.35 (1.77–3.12)	< 0.001
No (GAD-7 score < 10)	Ref		Ref	
**Depression**				
Yes (PHQ-9 score > 10)	1.45 (1.01–1.99)	0.015	1.35 (1.02–1.79)	0.035
No (PHQ-9 score < 10)	Ref		Ref	

### Characteristics of migraine

Almost 68% of the participants who had migraines reported the intensity of their headache as moderate to severe, and almost 88% of them had more than five attacks during the past month. Nausea was the most common symptoms associated with migraine (81%) followed by photophobia (67%) and vomiting (52.5%). More than 77% of the migraineurs had unilateral headache and pulsating in nature (85%). Almost 57.5% of the people with migraine had used analgesic more than five times during the past month and almost 42% had visited healthcare facilities due to their headache at least once during the last 12 months. Stress was the most commonly reported trigger of migraine among the participants (71%) followed by irregular sleep (47%), much reading (33%), noise (28%) and overuse of electronic device (25%) (Table 3).

**Table 3:** Headache related characteristics among the participants with migraine (*n* = 503)

Characteristics	N	%
**Intensity of headache**		
Mild	163	32.4
Moderate	185	36.8
Severe	155	30.8
**Frequency of headache during past month**		
0–5	111	22.1
6–10	184	36.6
11–15	137	27.2
> 15	71	14.1
**Associated symptoms**		
Nausea	407	80.9
Vomiting	264	52.5
Photophobia	337	67.0
**Characteristic of headache**		
Unilateral	389	77.3
Bilateral	114	22.7
Pulsating	428	85.1
Throbbing	75	14.9
**Frequency of analgesic use during the past month**		
0–5	214	42.5
6–10	155	30.8
11–15	99	19.7
> 15	35	7.0
**Frequency of healthcare facility visit during the past 12 months**		
Never	293	58.3
Once	110	21.9
Twice	72	14.3
3 times	17	3.4
> 3 times	11	2.2
**Migraine triggers (as reported by the participants in open ended question)**		
Stress	356	70.8
Irregular sleep	237	47.1
Academic pressure	167	33.2
External noise (e.g. loudspeaker, crowd etc.)	142	28.2
Electronic device use (e.g. laptop, mobile phones?	124	24.7
Physical activity	89	17.7
Exposure to sun	74	14.7
Menstruation in female	63	12.5
Smoking	46	9.1
Specific food or drink (e.g. coffee, tea, chocolates etc.)	38	7.6
Others	56	11.1

### Impact of migraine

**Fig. 1:**
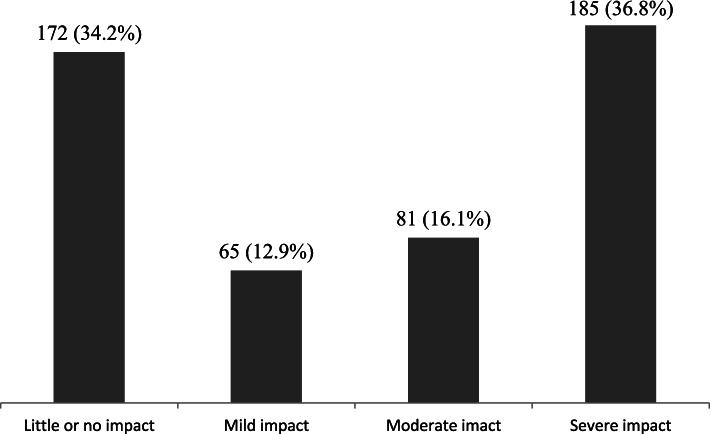
mpact of migraine among the participants (*n* = 503)

Almost 37% of the participants who had migraines reported that headache caused severe impact in their day to day activities (HIT-6 score ≥ 60), while mild and moderate levels of impact due to migraines were reported as 13% and 16% respectively. Only 34% of the people with migraine reported little or no impact due to migraine (HIT-6 score ≤ 49) (Fig. 1).

## Discussion

The present study was one of the primary attempts to determine the prevalence of migraine among the university students of Bangladesh. According to our findings, overall 21.4% of the university students were suffering from migraines, as screened by the self-reported ID Migraine™ tool. This prevalence was comparatively higher than the prevalence among the university students of neighboring India (14.12%) [9]. A meta-analysis of 56 studies including 34,904 university students reported that the prevalence of migraine ranges between 2.4% and 48.5% in different countries of the world. The pooled prevalence of migraine in university students was 16.1% (95% CI 13.6%–18.9%) [5].

The prevalence of migraine varies a lot according to the applied diagnostic criteria. For example, according to ICHD-1 criteria, the prevalence of migraine was 12.7%, according to ICHD-2 criteria it was 17.5%, while according to ICHD-3 criteria it was 29.2% [5]. Similarly, our used screening tool, ID Migraine™, reported the prevalence of migraine ranging from 12.2 to 28.1% with a combined prevalence of 18.9% [5]. The prevalence in our study population is consistent with the previous study findings using the same tool in different settings. The findings of the study should be interpreted considering the fact that the first wave of COVID-19 pandemic and country-wide lockdown was started during the data collection period, though the study design did not include the effect of the pandemic. The more recent studies evidenced that disease progression of migraine has been worsen during the lockdown and pandemic due to negative behavioral factors like pandemic related psychological distress, poor sleep quality and home confinement [26–28].

The prevalence of migraine was significantly higher among female participants of our study (29% in female and 12% in male). Migraine was reported as more prevalent among females in many of the previous studies [29–31]. However, some studies reported no significant difference between males and females [32, 33]. This gender difference in a conservative setting like Bangladesh needs further exploration. According to our findings, participants from the higher socioeconomic conditions were more likely to suffer from migraines (27%). This finding is contradictory with the social causation hypothesis of migraine as most of the previous studies reported lower socioeconomic conditions as a risk factor of migraine [34–37]. Further investigations using a well-established clinician-administered diagnostic tool may resolve the ambiguity.

Among the lifestyle-related factors, infrequent exercise (moderate exercise for at least 30 min for less than three days a week) and higher screen time (> 6 h) were associated with migraine. However, regular exercise (3 or more days a week) was not associated with migraine in logistic regression in our study. Though irregular exercise can trigger migraine attacks, there are pieces of evidence that regular exercise may have a prophylactic effect on migraine frequency which is most probably due to an altered migraine triggering threshold in persons who exercise regularly [38–40]. Similar to our findings, higher screen time showed a positive association with migraine prevalence in some previous studies [41, 42]. However, during the lockdown period due to COVID-19 pandemic, work from home strategy made people use screen for abnormally long period of time. It has deteriorate the daily lifestyle of people, caused poor sleep, and psychological distress which is evidenced to increase headache attacks in people with migraine [27, 28]. Other lifestyle-related factors such as tobacco smoking and substance abuse which have been reported as risk factors of migraines in previous studies [43–45] were not found to be associated with migraines in our study.

Symptoms of anxiety and depression were significantly associated with migraine among our study population. The risk of having migraines was more among the participants with anxiety compared to those with depressive symptoms, which is consistent with a previous finding [46]. There are a number of existing evidence supporting the association between migraine and psychological distress [47–49]. However, due to the cross-sectional nature of the study, the relation between migraine and psychological distress remained ambiguous. Moreover, anxiety and depressive symptoms might be over-estimated as there are some recent studies reporting that during the pandemic a huge number of university students faced these problems [50–52].

Elevated stress level, regular sleep disturbance, academic pressure and external noise were the most frequently reported triggers of migraine among our participants, which is consistent with the finding of a review article including 25 studies where these particular factors were enlisted among the top ten triggering factors of migraine [4]. One-third of the participants with migraines reported more than ten attacks of migraines during the past month and almost similar percentages of them reported severe headaches during the attacks. This high rate of frequency and intensity of headache during the migraine attacks caused severe migraine-related disability of more than 37% of the people with migraine as measured by the HIT-6 scale. This finding is consistent with the findings of some previous studies conducted using the same scale or the MIDAS scale [53, 54], though some other studies reported a higher rate of severe disability compared to our findings (55, 56).

### Strengths and limitations

The present study provides baseline information about the prevalence, associated factors, and day-to-day impact of migraine among the university students of Bangladesh. We anticipate that the findings will be somehow helpful for understanding the epidemiology of migraines among students in this country and guide further research on the people with migraine. However, some limitations of the study would be worth mentioning. Firstly, the study was conducted in a specific cohort of the population (university students), so the findings cannot be inferential for the overall population. Moreover, an online survey based on convenient sampling had a potential risk of sampling bias, which could influence the accuracy of the findings. Secondly, convenient sampling might create response bias where people of interest might be more responsive to the survey and it might result in over-estimation of the prevalence. Being a self-administered survey under-reporting or over-reporting behavior of the participants as well as recall bias could not be rolled out. Thirdly,, being a cross-sectional study, it had failed to establish any causal relationship between migraine and the dependent variables. And most importantly, we used ID Migraine™, a self-administered screening tool for migraine, which was not clinically diagnostic. Finally, the data for the study was collected during initial lockdown period of COVID-19 pandemic which might limit the findings from generalizability. Further studies using clinical diagnostic criteria under the supervision of the clinicians is suggested to get a more comprehensive insight into the epidemiology of migraines in Bangladesh.

## Conclusions

Our study shows that the prevalence of migraine among university students of Bangladesh is alarmingly high, especially female students suffer more. Several modifiable factors including lack of exercise, high screen time and symptoms of anxiety and depression are associated with migraines. Frequent migraine attacks and severe intensity of headache cause a substantial level of disability among these young sufferers. Cautious avoidance of the triggering factors through appropriate interventions and prophylactic medication can mitigate the negative impact of migraine as well as improve the quality of life. We suggest further large-scale longitudinal studies using standard clinical diagnostic tools to report nationwide prevalence & factors associated to understand the epidemiology of migraine among students in Bangladesh.

## Data Availability

Data will be available on request to the corresponding author.

## Abbreviations

AOR: Adjusted Odds Ratio
CHERRIES: Checklist for Reporting Results of Internet ESurveys
CI: Confidence interval
GAD: Generalized Anxiety Disorder
HIT: Headache Impact Test
IBM: International Business Machines
ICHD: International Classification of Headache Disorders
MIDAS: Migraine Disability Assessment
PHQ: Patient Health Questionnaire
PSQI: Pittsburgh sleep quality index
RMCH: Rajshahi Medical College & Hospital; SE- Standard error
SPSS: Statistical Package for the Social Sciences

## References

[1] James SL, Abate D, Hassen Abate K, Abay SM, Abbafati C, Abbasi N, et al. Global, regional, and national incidence, prevalence, and years lived with disability for 354 diseases and injuries for 195 countries and territories, 1990â€“2017: a systematic analysis for the Global Burden of Disease Study 2017 [Internet]. 2018 [cited 2020 Sep 16]. Available from: https://github.com/ihmeuw/10.1016/S0140-6736(18)32279-7 PMC6227754 30496104

[2] Leonardi M, Raggi A (2013). Burden of migraine: International perspectives. Neurol Sci.

[3] Pesa J, Lage MJ (2004). The Medical Costs of Migraine and Comorbid Anxiety and Depression. Headache J Head Face Pain.

[4] Peroutka SJ. What Turns on a Migraine? A Systematic Review of Migraine Precipitating Factors [Internet]. Vol. 18, Current Pain and Headache Reports. Current Medicine Group LLC 1. 2014 [cited 2020 Sep 16]. p. 454. Available from: http://link.springer.com/10.1007/s11916-014-0454-z 10.1007/s11916-014-0454-z 25160711

[5] Wang X, Zhou HB, Sun JM, Xing YH, Zhu YL, Zhao YS. The prevalence of migraine in university students: A systematic review and meta-analysis [Internet]. Vol. 23, European Journal of Neurology. Blackwell Publishing Ltd. 2016 [cited 2020 Sep 16]. p. 464–75. Available from: https://onlinelibrary.wiley.com/doi/abs/10.1111/ene.12784 10.1111/ene.12784 26283142

[6] Al-Hashel JY, Ahmed SF, Alroughani R, Goadsby PJ (2014). Migraine among medical students in Kuwait University. J Headache Pain.

[7] Menon B, Kinnera N (2013). Prevalence and characteristics of migraine in medical students and its impact on their daily activities. Ann Indian Acad Neurol.

[8] Perveen I, Parvin R, Saha M, Bari MS, Huda MN, Ghosh MK (2016). Prevalence of Irritable Bowel Syndrome (IBS), Migraine and Co-Existing IBS-Migraine in Medical Students. J Clin DIAGNOSTIC Res.

[9] Ray B, Paul N, Hazra A, Das S, Ghosal M, Misra A, et al. Prevalence, burden and risk factors of migraine: A community-based study from Eastern India. Neurol India. 2017;65(6):1280 http://www.neurologyindia.com/text.asp?2017/65/6/1280/217979. 10.4103/0028-3886.217979 29133701

[10] Eysenbach G. Improving the quality of web surveys: The Checklist for Reporting Results of Internet E-Surveys (CHERRIES) [Internet]. Vol. 6, Journal of Medical Internet Research. Journal of Medical Internet Research; 2004 [cited 2020 Jul 19]. Available from: /pmc/articles/PMC1550605/?report=abstract 10.2196/jmir.6.3.e34 PMC1550605 15471760

[11] Mollayeva T, Thurairajah P, Burton K, Mollayeva S, Shapiro CM, Colantonio A. The Pittsburgh sleep quality index as a screening tool for sleep dysfunction in clinical and non-clinical samples: A systematic review and meta-analysis. Sleep Med Rev. 2016;23:52–73. 10.1016/j.smrv.2015.01.009 26163057

[12] Bicakci S, Bozdemir N, Over F, Saatci E, Sarica Y. Prevalence of migraine diagnosis using ID Migraine among university students in southern Turkey. J Headache Pain 2008;9(3):159–63. 10.1007/s10194-008-0031-0. 10.1007/s10194-008-0031-0 PMC3476199 18427728

[13] Lipton RB, Dodick D, Sadovsky R, Kolodner K, Endicott J, Hettiarachchi J (2003). A self-administered screener for migraine in primary care: The ID migraine^TM^ validation study. Neurology.

[14] Cousins G, Hijazze S, Van De Laar FA, Fahey T (2011). Diagnostic accuracy of the ID migraine: A systematic review and meta-analysis. Headache.

[15] Tfelt-Hansen P, Pascual J, Ramadan N, Dahlöf C, D’Amico D, Diener H-C (2012). Guidelines for controlled trials of drugs in migraine: third edition A guide for investigators. Cephalalgia.

[16] Kosinski M, Bayliss MS, Bjorner JB, Ware JEJ, Garber WH, Batenhorst A (2003). A six-item short-form survey for measuring headache impact: The HIT-6. Qual Life Res.

[17] Yang M, Rendas-Baum R, Varon SF, Kosinski M (2011). Validation of the headache impact test (HIT-6^TM^) across episodic and chronic migraine. Cephalalgia.

[18] Spitzer RL, Kroenke K, Williams JBW (1999). Validation and utility of a self-report version of PRIME-MD: the PHQ primary care study. J Am Med Assoc.

[19] Islam S, Akter R, Sikder T, et al. Prevalence and Factors Associated with Depression and Anxiety Among First-Year University Students in Bangladesh: A Cross-Sectional Study. Int J Ment Health Addiction. 2020. 10.1007/s11469-020-00242-y.

[20] Islam MS, Rahman ME, Moonajilin MS, Griffiths MD. Validation and evaluation of the psychometric properties of bangla nine-item Internet Disorder Scale–Short Form. J Addict Dis. 2020;38(4):540–549. 10.1080/10550887.2020.1799134 32762512

[21] Kroenke K, Spitzer RL, Williams JBW (2001). The PHQ-9: Validity of a brief depression severity measure. J Gen Intern Med.

[22] Spitzer RL, Kroenke K, Williams JBW, Löwe B (2006). A brief measure for assessing generalized anxiety disorder: The GAD-7. Arch Intern Med.

[23] Islam MS, Ferdous MZ, Potenza MN (2020). Panic and generalized anxiety during the COVID-19 pandemic among Bangladeshi people: An online pilot survey early in the outbreak. J Affect Disord.

[24] Moonajilin MS, Rahman ME, Islam MS (2020). Relationship between overweight/obesity and mental health disorders among Bangladeshi adolescents: A cross-sectional survey. Obes Med.

[25] Chan YH. Biostatistics 202: logistic regression analysis. Singapore Med J. 2004;45(4):149–53. 15094982

[26] Gentile E, Delussi M, Abagnale C, Caponnetto V, De Cesaris F, Frattale I, et al. Migraine during COVID-19: Data from Second Wave Pandemic in an Italian Cohort. Brain Sci. 2021;11(4). Available from: https://pubmed.ncbi.nlm.nih.gov/33920175/10.3390/brainsci11040482 PMC8070557 33920175

[27] Di Stefano V, Ornello R, Gagliardo A, Torrente A, Illuminato E, Caponnetto V, et al. Social distancing in chronic migraine during the covid-19 outbreak: Results from a multicenter observational study. Nutrients. 2021;13(4). Available from: /labs/pmc/articles/PMC8074143/10.3390/nu13041361 PMC8074143 33921674

[28] Currò CT, Ciacciarelli A, Vitale C, Vinci ES, Toscano A, Vita G, et al. Chronic migraine in the first COVID-19 lockdown: the impact of sleep, remote, working and other life/psychological changes. Neurol Sc. 2021;42(11):4403–18 https://pubmed.ncbi.nlm.nih.gov/34365547/. 10.1007/s10072-021-05521-7 PMC8349308 34365547

[29] Seifert T, Sufrinko A, Cowan R, Scott Black W, Watson D, Edwards B (2017). Comprehensive headache experience in collegiate student-athletes: an initial report from the NCAA headache task force. Headache.

[30] Birru EM, Abay Z, Abdelwuhab M, Basazn A, Sirak B, Teni FS. Management of headache and associated factors among undergraduate medicine and health science students of University of Gondar, North West Ethiopia. J Headache Pain. 2016;17:56. 10.1186/s10194-016-0647-4. 10.1186/s10194-016-0647-4 PMC4877336 27216280

[31] Lebedeva ER, Kobzeva NR, Gilev D V., Olesen J. Factors Associated with Primary Headache According to Diagnosis, Sex, and Social Group. Headache. 2016;56(2):341–56. Available from: https://pubmed.ncbi.nlm.nih.gov/26833220/10.1111/head.12757 26833220

[32] Wang X, Sun J, Xing Y, Zhou H, Zhao Y, Zhu Y (2015). The prevalence and awareness of migraine among university students in Harbin. China. J Oral Facial Pain Headache.

[33] Yu S, Liu R, Zhao G, Yang X, Qiao X, Feng J (2012). The prevalence and burden of primary headaches in China: A population-based door-to-door survey. Headache.

[34] Stewart WF, Roy J, Lipton RB (2013). Migraine prevalence, socioeconomic status, and social causation. Neurology.

[35] Bigal ME, Lipton RB, Winner P, Reed ML, Diamond S, Stewart WF. Migraine in adolescents: Association with socioeconomic status and family history. Neurology. 2007;69(1):16–25. Available from: https://n.neurology.org/content/69/1/16 10.1212/01.wnl.0000265212.90735.64 17606878

[36] Bigal ME, Lipton RB, Winner P, Reed ML, Diamond S, Stewart WF. Migraine in adolescents: Association with socioeconomic status and family history. Neurology. 2007;69(1):16–25. Available from: https://n.neurology.org/content/69/1/16 10.1212/01.wnl.0000265212.90735.64 17606878

[37] Winter AC, Berger K, Buring JE, Kurth T. Associations of socioeconomic status with migraine and non-migraine headache. Cephalalgia. 2012;32(2):159–70. Available from: http://journals.sagepub.com/doi/10.1177/0333102411430854 10.1177/0333102411430854 PMC3266434 22174348

[38] Amin FM, Aristeidou S, Baraldi C, Czapinska-Ciepiela EK, Ariadni DD, Di Lenola D, et al. The association between migraine and physical exercise [Internet]. Vol. 19, The journal of headache and pain. BioMed Central; 2018 [cited 2020 Sep 22]. p. 83. Available from: 10.1186/s10194-018-0902-y 10.1186/s10194-018-0902-y PMC6134860 30203180

[39] Ahn AH (2013). Why does increased exercise decrease migraine?. Curr Pain Headache Rep..

[40] Daenen L, Varkey E, Kellmann M, Nijs J. Exercise, Not to Exercise, or How to Exercise in Patients With Chronic Pain? Applying Science to Practice. Clin J Pain. 2015;31(2):108–14. Available from: http://journals.lww.com/00002508-201502000-00003 10.1097/AJP.0000000000000099 24662498

[41] Malkki H. Migraine: Long screen time exposure could increase the risk of migraine [Internet]. Vol. 12, Nature Reviews Neurology. Nature Publishing Group. 2016:4. Available from: http://dx.doi.org/10.1177/0333102415620286 10.1038/nrneurol.2015.238 26678985

[42] Montagni I, Guichard E, Carpenet C, Tzourio C, Kurth T (2016). Screen time exposure and reporting of headaches in young adults: A cross-sectional study. Cephalalgia.

[43] Panconesi A. Alcohol and migraine: Trigger factor, consumption, mechanisms. A review [Internet]. Vol. 9, Journal of Headache and Pain. BioMed Central. 2008:19–27. Available from: https://thejournalofheadacheandpain.biomedcentral.com/articles/10.1007/s10194-008-0006-1 10.1007/s10194-008-0006-1 PMC3476173 18231712

[44] Rozen TD (2011). A history of cigarette smoking is associated with the development of cranial autonomic symptoms with migraine headaches. Headache J Head Face Pain.

[45] Aamodt AH, Stovner LJ, Hagen K, Bråthen G, Zwart J (2006). Headache prevalence related to smoking and alcohol use. The Head-HUNT study. Eur J Neurol.

[46] Peres MFP, Mercante JPP, Tobo PR, Kamei H, Bigal ME. Anxiety and depression symptoms and migraine: a symptom-based approach research. J Headache Pain. 2017;18(1). Available from: https://pubmed.ncbi.nlm.nih.gov/28324317/10.1186/s10194-017-0742-1 PMC5360747 28324317

[47] Minen MT, De Dhaem OB, Van Diest AK, Powers S, Schwedt TJ, Lipton R, et al. Migraine and its psychiatric comorbidities. Vol. 87, Journal of Neurology, Neurosurgery and Psychiatry. BMJ Publishing Group. 2016:741–9. Available from: https://pubmed.ncbi.nlm.nih.gov/26733600/10.1136/jnnp-2015-312233 26733600

[48] Song TJ, Cho SJ, Kim WJ, Yang KI, Yun CH, Chu MK (2017). Anxiety and depression in probable migraine: A population-based study. Cephalalgia.

[49] Demir UF, Bozkurt O. Effects of perceived social support, depression and anxiety levels on migraine. Arch Neuropsychiatry. 2020;57(3). Available from: https://pubmed.ncbi.nlm.nih.gov/32952423/10.29399/npa.25000 PMC7481976 32952423

[50] AkhtarulIslam M, Barna SD, Raihan H, NafiulAlamKhan M, TanvirHossain M (2020). Depression and anxiety among university students during the COVID-19 pandemic in Bangladesh: A web-based cross-sectional survey. PLoS One.

[51] Khan AH, Sultana MS, Hossain S, Hasan MT, Ahmed HU, Sikder MT (2020). The impact of COVID-19 pandemic on mental health & wellbeing among home-quarantined Bangladeshi students: A cross-sectional pilot study. J Affect Disord.

[52] Safa F, Anjum A, Hossain S, Trisa TI, Alam SF, Abdur Rafi M (2021). Immediate psychological responses during the initial period of the COVID-19 pandemic among Bangladeshi medical students. Child Youth Serv Rev.

[53] Shin HE, Jeong ;, Park W, Yeong ;, Kim I, Kwang ;, et al. Headache Impact Test-6 (HIT-6) Scores for Migraine Patients: Their Relation to Disability as Measured from a Headache Diary. J Clin Neurol. 2008;4:158–63. Available from: www.headachetest.com/HIT6translations.html 10.3988/jcn.2008.4.4.158 PMC2686853 19513291

[54] Goldstein ED, Badi MK, Klaas JP, Glover P, Rozen TD, Huang JF, et al. A Cross-Sectional Analysis of Migraine-Related Disability in CADASIL: A Mayo Clinic Cohort. Neurologist. 2019;24(6):161–4. Available from: https://pubmed.ncbi.nlm.nih.gov/31688705/10.1097/NRL.0000000000000253 31688705

[55] Alemayehu Ayele B, Mamushet Yifru Y. Migraine-related disability and co-morbid depression among migraineurs in Ethiopia: a cross-sectional study. 10.1186/s12883-018-1095-3 10.1186/s12883-018-1095-3 PMC6027576 29966529

[56] Yang M, Rendas-Baum R, Varon SF, Kosinski M (2011). Validation of the Headache Impact Test (HIT-6^TM^) across episodic and chronic migraine. Cephalalgia.

